# Supplementary material for: Overcoming strength-ductility tradeoff with high pressure thermal treatment
Source: Nat Commun. 2024 May 10;15:3932. doi: 10.1038/s41467-024-48435-6 (PMC11087546; doi:10.1038/s41467-024-48435-6)
Supplement: Supplementary file 1 — Supplementary Information [file 41467_2024_48435_MOESM1_ESM.pdf]

Supplementary information for

Overcoming strength-ductility tradeoff with high pressure thermal treatment

Yao Tang[1,2], Haikuo Wang[1*], Xiaoping Ouyang[1,3*], Chao Wang[1], Qishan Huang[2], Qingkun Zhao[2], Xiaochun Liu[4], Qi Zhu[5], Zhiqiang Hou[1], Jiakun Wu[1], Zhicai Zhang[1], Hao Li[1], Yikan Yang[1], Wei Yang[2], Huajian Gao[5,6,7*], Haofei Zhou[2*]

[1]Center for High Pressure Science and Technology, College of Energy Engineering, Zhejiang University, Hangzhou, China

[2]State Key Laboratory of Fluid Power and Mechatronic Systems, Center for X-Mechanics, Department of Engineering Mechanics, Zhejiang University, Hangzhou, China

[3]School of Materials Science and Engineering, Xiangtan University, Xiangtan, China

[4]Institute of Metals, College of Material Science and Engineering, Changsha University of Science and Technology, Changsha, China

[5]School of Mechanical and Aerospace Engineering, College of Engineering, Nanyang Technological University, Singapore 639798, Singapore

[6]Institute of High Performance Computing, A*STAR, Singapore 138632, Singapore

* Corresponding author.

Email: haofei_zhou@zju.edu.cn; gao.huajian@tsinghua.edu.cn; oyxp2003@aliyun.com; haikuo.wang@zju.edu.cn

This document contains Supplementary Figures (Supplementary Figs. 1-24), Supplementary Notes (1-6) and Supplementary References (1-37).

[7] Mechano-X Institute, Applied Mechanics Laboratory, Department of Engineering Mechanics, Tsinghua University, Beijing 100084, China

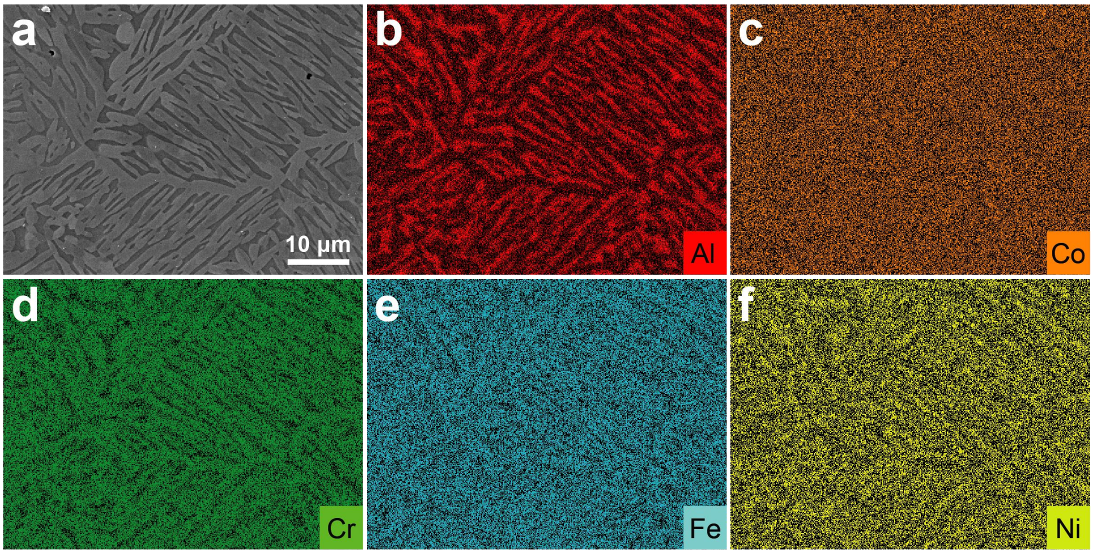

Supplementary Fig. 1 | Composition analysis of the homogenized (Ho) $Al_{0.7}$CoCrFeNi (molar ratio) high entropy alloy. a, Scanning electron microscope (SEM) image of the Ho $Al_{0.7}$CoCrFeNi alloy. **b-f,** The corresponding element mapping: Al, Co, Cr, Fe, Ni.

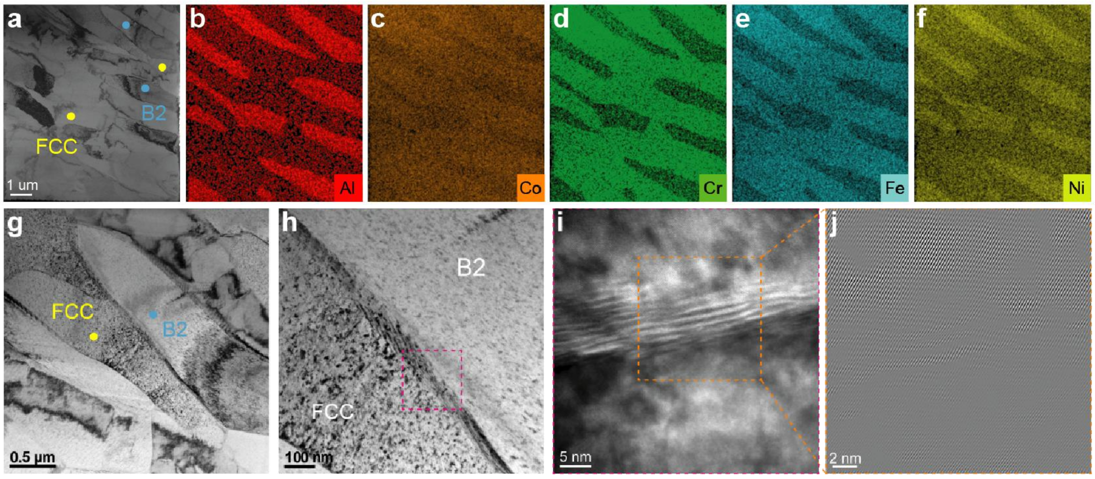

Supplementary Fig. 2 | Microstructure of the Ho $Al_{0.7}$CoCrFeNi high entropy alloy. a-f, Transmission electron microscopy (TEM) image of the Ho alloy and corresponding element mapping. **g,** Low magnification TEM image of the Ho alloy, showing the dual-phase structure. **h,** High magnification TEM image of the Ho alloy, showing the transition layer at the interface. **i** and **j,** Typical high-resolution TEM image and the corresponding inverse fast Fourier transform (IFFT) image of the interface region.

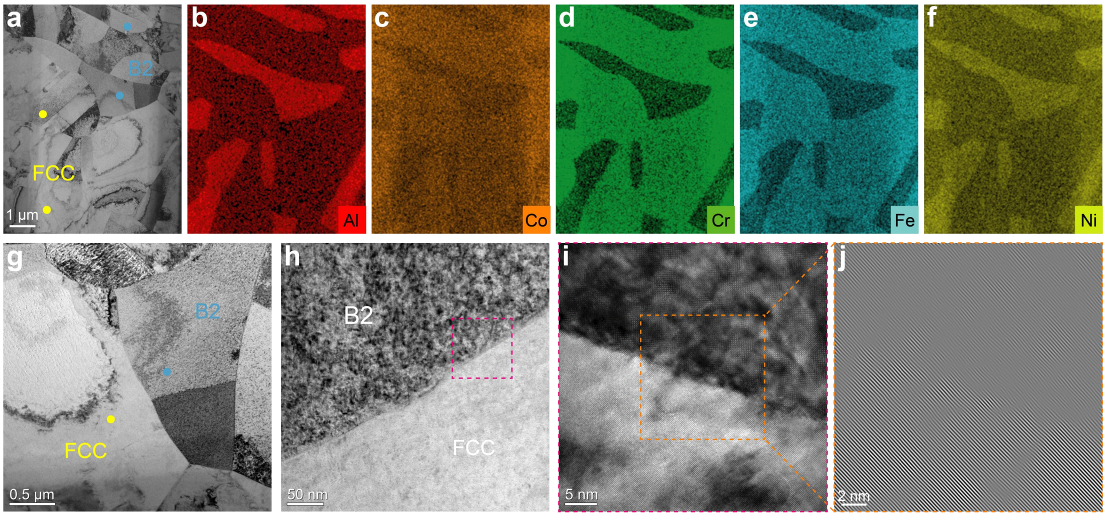

Supplementary Fig. 3 | Microstructure of the HPHT-treated $Al_{0.7}$CoCrFeNi high entropy alloy. a-f, TEM image of the HPHT-treated alloy and corresponding element mapping. **g,** Low magnification TEM image of the HPHT-treated alloy sample, showing the dual-phase structure. **h,** High magnification TEM image of the HPHT-treated alloy sample, showing the sharp interface. **i** and **j,** Typical HRTEM image and the corresponding IFFT image of the interface region.

Supplementary Note 1 | The comparison between high pressure thermal treatment and conventional hot isostatic pressing treatment.

The hot isostatic pressing (HIP) treatment has been used for upgrading castings, densifying presintered components, consolidating powders, and interfacial bonding[1]. It involves the simultaneous application of pressure and temperature in a specially constructed vessel. As shown in Supplementary Fig. 4a, the pressure is applied with a gas. The pressure of the HIP treatment is usually on the order of 100 MPa[2], which is much lower than that of our HPHT treatment (6 GPa). In HIP treatment, although the pressure is isostatic, shrinkage is not generally isotropic, particularly if containment is used[3]. The problem with using gas as the pressure medium for the HIP treatment lies in that the reduction in pore surface energy takes place during the HIP process and acts as a driving force for pore closure, while the entrapped gas backpressure inside the pore reacts adversely[3]. Once a pore starts to shrink through the HIP, the entrapped gas pressure boosts up inside the pores and prohibits further closure. Therefore, the closure of the inner pores was correlated with the trapped gas, and the pores with gas inside can hardly be closed during HIP. In our HPHT treatment, to avoid such problem, six WC anvils are fixed on six pistons and simultaneously pushed by six hydraulic cylinders to operate cubic press. The six anvils define a center cubic cavity, inside of which is the cubic assembly in our experiments, as shown in Supplementary Fig. 4b. The motion of the six anvils compresses the cubic assembly so that the sample chamber pressure continues to build up in the assembly with a decrease in the volume of the pyrophyllite cube. Such a combination of pressure transmission media can produce a quasi-hydrostatic stress state and obtain a fully densified sample.

Moreover, grain coarsening is a common phenomenon during the HIP process, having an unfavorable effect on mechanical properties that need to be considered[4]. Because of the very slow cooling rate associated with HIP treatment, the harmful phases may precipitate during the cooling step. For example, Joseph et al. observed the formation of σ phase in an $Al_{0.85}CoCrFeNi$ alloy produced by HIP treatment, such microstructural evolution resulted in a sharp loss in ductility[5]. Remarkably, the HPHT-treated alloy in our work demonstrated exceptional grain coarsening resistance with negligible grain growth. At the same time, the high solidification rates associated with the HPHT fabrication route do not allow sufficient time for the harmful phases to form.

Most importantly, the effect of HIP treatment on the microstructure and mechanical behavior

of HEAs is similar to those observed for traditional processing methods. For example, the HIP-processing of the $Al_{0.85}CoCrFeNi$ alloy resulted in the coarsening of the microstructure and resulted in the significant loss of ductility after processing by HIP[5]. At the same time, although the HIP can improve the ductility of high entropy alloy, it unfortunately reduces the strength[4]. These results suggest that HIP treatment still has limitations in overcoming the strength-ductility trade-off of structural materials. In the present work, to demonstrate the effect of HIP treatment on the mechanical property of the $Al_{0.7}CoCrFeNi$ alloy, hot isostatic pressing was carried out at 1473 K under 150 MPa for a holding time of 2 hours and then uniaxial quasi-static tensile tested to evaluate the mechanical property. Supplementary Fig. 5 shows the engineering stress-strain curves of the $Al_{0.7}CoCrFeNi$ alloy in both the homogenized and HIP-treated conditions (Ho+HIP) for tensile testing at room temperature. A noticeable increase in tensile strength occurs after the HIP treatment, the elongation decreases a little. By contrast, our HPHT-treated alloy exhibits nearly 100% enhancement in both strength and tensile ductility compared with the Ho alloy. The HPHT treatment can be used to achieve synergistic enhancement in strength and ductility compared with the HIP treatment. Such HPHT-induced strength-ductility synergy is attributed to the formation of a hierarchically patterned BCC/FCC microstructure with coherent interfaces, which promotes the activation of multiple deformation mechanisms at multiple length scales.

Overall, the proposed HPHT treatment is fundamentally different from the HIP treatment in terms of the processing method, adjustment of microstructure and impact on macroscopic mechanical properties. The HPHT treatment provides a novel and promising route for tailing the mechanical properties of structural materials.

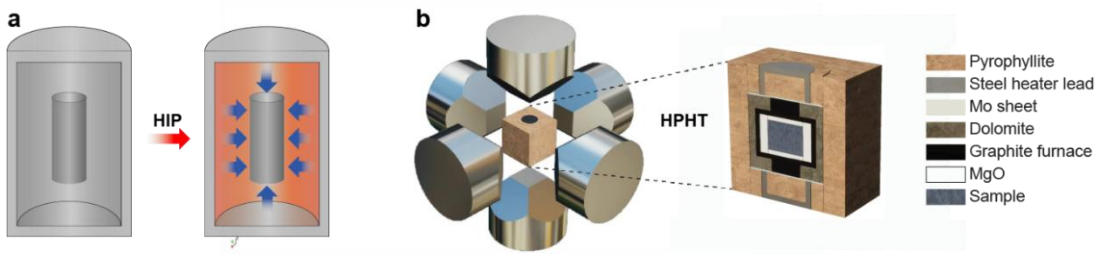

Supplementary Fig. 4 | The comparison between HPHT treatment and HIP treatment. a, Schematic diagram of the hot isostatic pressing (HIP) treatment. **b,** Schematic diagram of the HPHT treatment set-up and the sample assembly part.

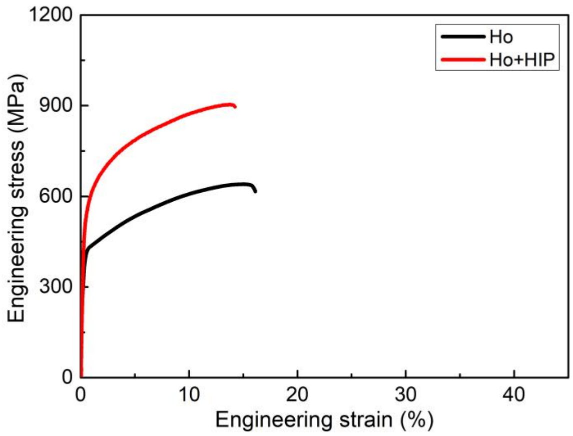

Supplementary Fig. 5 | True stress-strain curves of the homogenized alloy (Ho alloy) after HPHT treatment (Ho+HIP), along with the Ho alloy before HIP treatment.

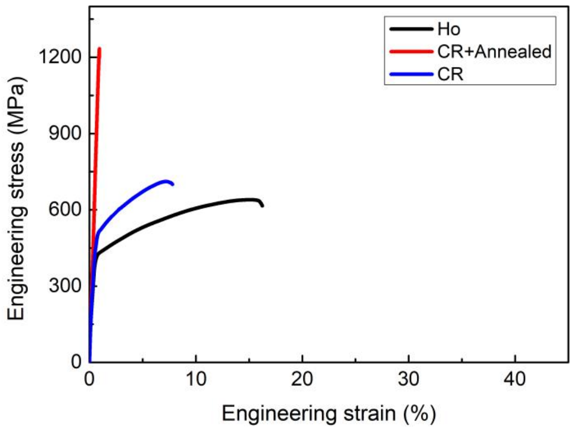

Supplementary Fig. 6 | True stress-strain curves of the homogenized alloy (Ho alloy), along with the cold-rolled alloy (CR alloy) and the CR+Annealed alloy.

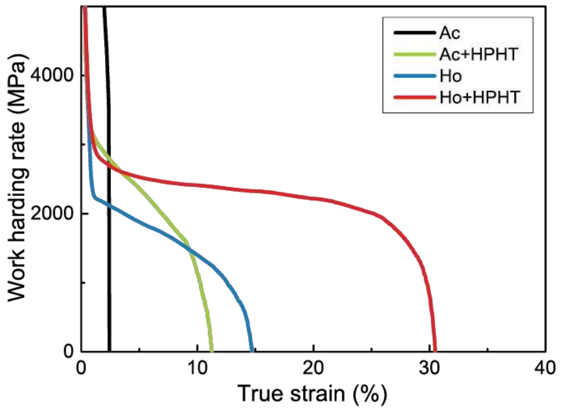

Supplementary Fig. 7 | Tensile properties at room temperature. Strain-hardening rate with respect to true strain.

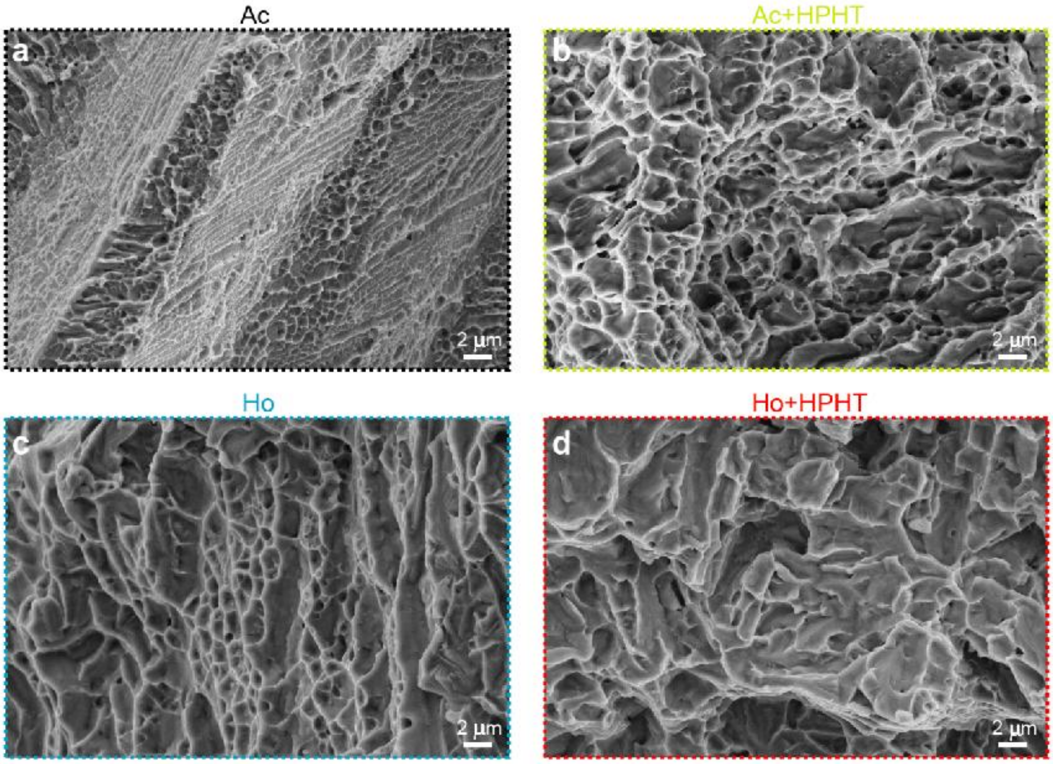

Supplementary Fig. 8 | Fracture morphology of the $Al_{0.7}CoCrFeNi$ high entropy alloys. a, Ac, **b,** Ac+HPHT, **c,** Ho, **d,** Ho+HPHT. A richly dimpled structure can be seen on the fracture surface of the HPHT-treated alloy compared to the Ho alloy. The hierarchically patterned microstructure consisting of hexagonal-like structural units can still be seen faintly in the fractured HPHT-treated alloy. These structures exhibit the characteristic of ductile fracture mode and indicate a superior plastic deformation ability of the HPHT-treated alloy. Compared with the brittle cleavage fracture mode of Ac alloy, the ductile fracture mode of Ac+HPHT alloy can be observed.

Supplementary Note 2 | Microstructures and mechanical properties of the $Al_{0.7}CoCrFeNi$ high entropy alloys treated at different HPHT conditions

We note that the HPHT-treated alloy reported in this work was designed to demonstrate the proposed HPHT strategy. In alloy modification, the microstructures of the HEAs depend on the variation and distribution of two phases, which is substantially influenced by the HPHT conditions. Here, we purposely adjust the pressure and temperature conditions during HPHT treatment to engineer the phase distribution. In addition to the HPHT-treated alloy treated at 1474 K, we also treated the alloy at 1373 K and 1573 K, which are denoted as HPHT-treated-1373K and HPHT-treated-1573K, respectively. The microstructures and mechanical properties of the alloys treated at different HPHT conditions are presented in Supplementary Fig. 9 to show the influence of the HPHT conditions. The corresponding electron backscatter diffraction (EBSD) phase map of the HPHT-treated-1373K alloy reveals a phase arrangement pattern in which the polygon structural units are interconnected by the FCC phase. The volume fractions of FCC and BCC phases in the HPHT-treated-1373K alloy were determined by EBSD to be ~ 69% and ~ 31%, nearly identical to those in the Ho alloy (~ 68% and ~ 32%). Although the polygon-like structure in HPHT-treated-1373K alloy is not as regular as the hexagonal structure in HPHT-treated-1473K alloy, the specific structure caused by HPHT can already provide substantial property enhancement compared to the Ho alloy. For the HPHT-treated-1573K alloy, obvious grain growth and phase segregation can be seen in Supplementary Fig. 9. The volume fractions of FCC and BCC phases in the HPHT-treated-1573K alloy were determined by EBSD to be ~ 58% and ~ 42%. The segregation and the high content of the BCC phase will lead to a decrease in mechanical properties. Although the mechanical properties have slightly decreased compared to that of HPHT-treated-1473K alloy, they are still significantly improved compared to the Ho alloy. These results indicate that the HPHT conditions can be tuned to modify the microstructure of the $Al_{0.7}CoCrFeNi$ alloy.

These results indicate that the HPHT conditions can be tuned to modify the microstructure of the treated material ($Al_{0.7}CoCrFeNi$ in the present study). More specifically, the temperature can influence grain growth and affect the distribution of phases. When the HPHT treatment temperature is relatively low, the pressure becomes the dominating factor that limits the formation of hierarchically patterned microstructure. On the other hand, when the HPHT treatment

temperature is relatively high, significant grain growth occurs and the content of hard phases increases. These trends indicate the existence of an optimum temperature for the best mechanical properties. With the optimal temperature of HPHT treatment, the corresponding microstructure transforms from lamellae to a hierarchically patterned microstructure. In particular, the hexagonal-like structural units might be attributed to the hexahedral press we used and the fact that the grains themselves tend to be a hexagonal-like structure in the densest and relatively steady state. The hierarchically patterned microstructure consisting of hexagonal-like structural units can make the permeating arrangement between the two phases more obvious, and the mutual constraints between the two phases stronger, thus endowing the structure with higher stability[6]. On the other hand, high-pressure annealing effectively offsets the driving force for grain coarsening[7-9], which promotes the change of incoherent interfaces to coherent ones. The latter would decrease the interface energy for further stabilizing the hierarchically patterned microstructure.

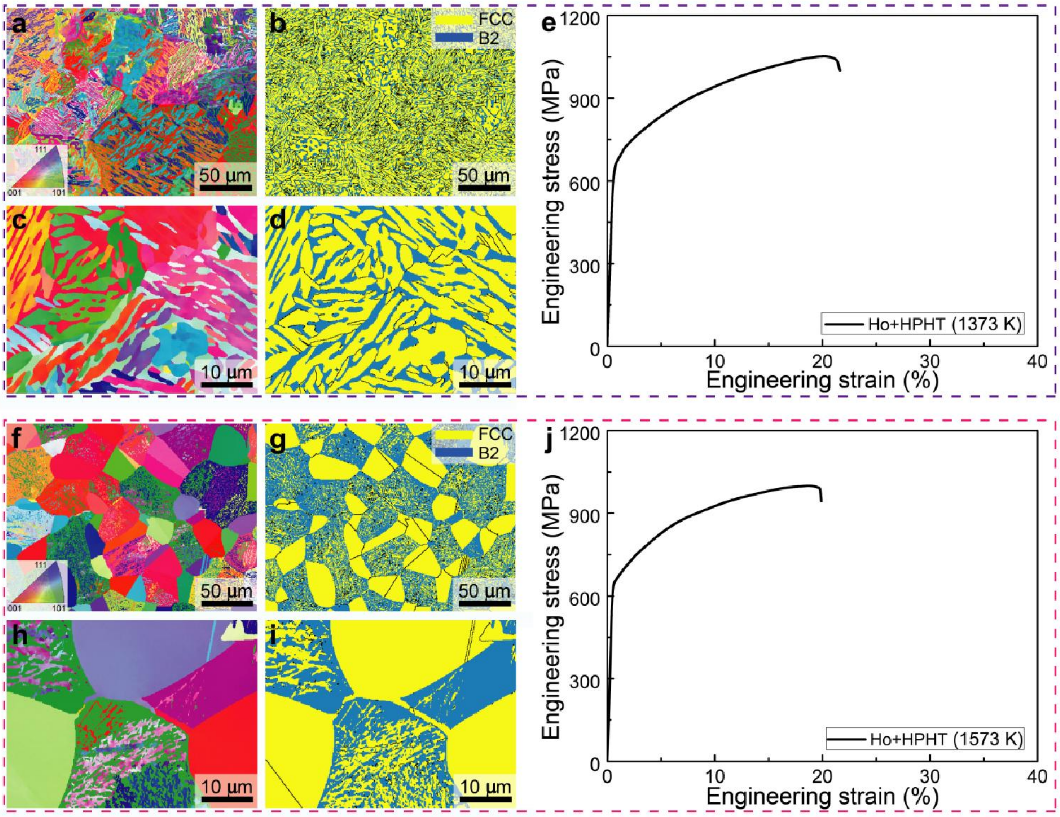

Supplementary Fig. 9 | The effect of HPHT treatment conditions on the structure and mechanical properties of the $Al_{0.7}$CoCrFeNi alloy. a-d, Low and high magnifications of the electron backscatter diffraction (EBSD) inverse-pole figure (IPF) maps and corresponding EBSD phase maps in the $Al_{0.7}$CoCrFeNi alloy treated at 6 GPa and 1373 K. **e,** Engineering stress-strain curve of the $Al_{0.7}$CoCrFeNi alloy treated at 6 GPa and 1373 K. **f-i,** Low and high magnifications of the electron backscatter diffraction (EBSD) inverse-pole figure (IPF) maps and corresponding EBSD phase maps in the $Al_{0.7}$CoCrFeNi alloy treated at 6 GPa and 1573 K. **j,** Engineering stress-strain curve of the $Al_{0.7}$CoCrFeNi alloy treated at 6 GPa and 1573 K.

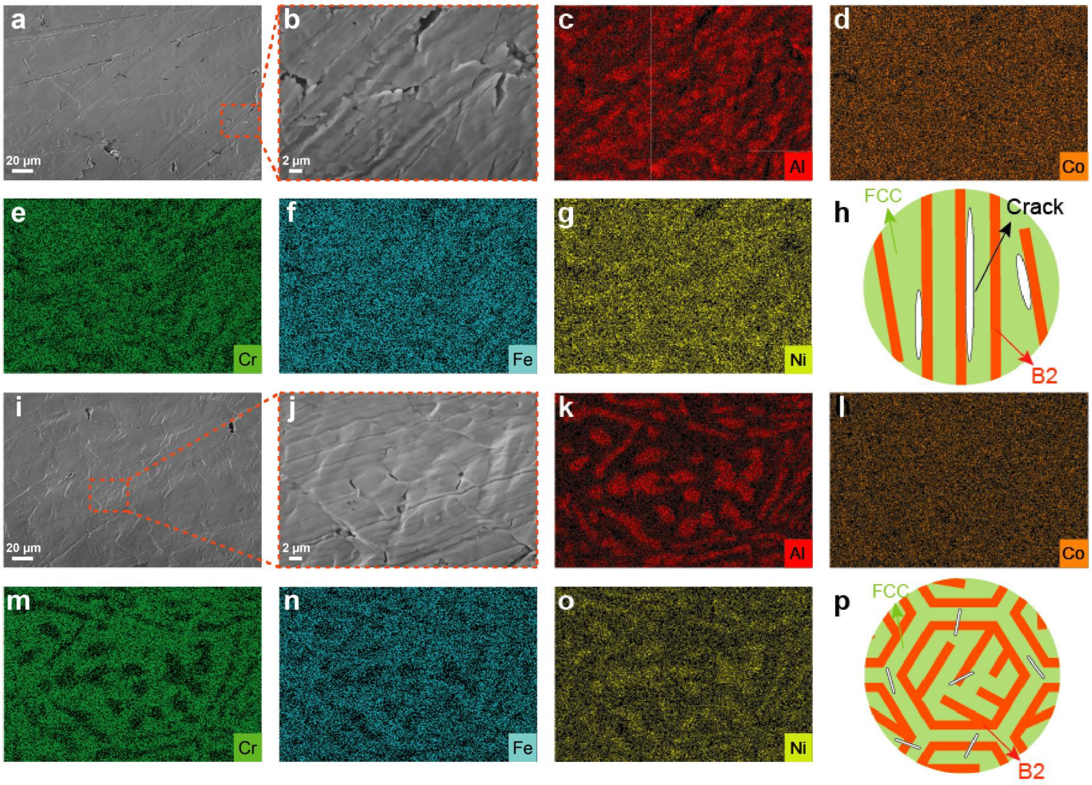

Supplementary Fig. 10 | The crack behavior of the Ho and HPHT-treated $Al_{0.7}CoCrFeNi$ alloys. a, SEM secondary electron (SE) image of the fracture surface in the Ho alloy. **b,** An enlarged image of the fracture surface in the Ho alloy showing the long cracks. **c-g,** The corresponding element mapping for the enlarged image of the fracture surface in the Ho alloy. **h,** Schematic diagram illustrating the crack behaviors in the fractured Ho alloy. **i,** SEM secondary electron (SE) image of the fracture surface in the HPHT-treated alloy. **j,** An enlarged image of the fracture surface in the HPHT-treated alloy showing numerous isolated micro-cracks distributed in the FCC and BCC phases. Some micro-cracks cut through the B2 phase. **k-o,** The corresponding element mapping for the enlarged image of the fracture surface in the HPHT-treated alloy. **p,** Schematic diagram illustrating the crack behaviors in the fractured HPHT-treated alloy.

Supplementary Note 3 | Microstructures and crack behaviors of the Ac $Al_{0.7}CoCrFeNi$ and Ac+HPHT alloys

The Ho $Al_{0.7}CoCrFeNi$ alloy was chosen as the precursor material for HPHT treatment. The Ho alloy features structural uniformity with soft/hard phases instead of bimodal grains in an as-cast state. As shown in Supplementary Fig. 11b, the EBSD phase map reveals that the dual-phase lamellae consist of alternating FCC layers and BCC layers in the Ac alloy. The spatial distribution of FCC and BCC phases is uneven and there is a segregation phenomenon. With the HPHT treatment at 1473 K for 2 h, the distribution of FCC and BCC phases becomes more uniform (Supplementary Fig. 11j). More importantly, the lamellae dual phase structure of Ac alloy can also be remarkably modified into a more restrictive structure with polygon-like structural units by HPHT treatment. These changes in the microstructure resulted in the improvement of tensile ductility in the HPHT-treated alloy and the reduction of strength. The Ac sample has a coarse columnar structure and significant B2 phase segregation. The agglomerated B2 region has limited the plastic deformability, which can be an obstacle to the strengthening of Ac sample. At the same time, the B2 phase has no tensile ductility, due to the instability of the B2 phase to accommodate shape changes[10]. For the Ac sample, the high HPHT treatment involves annealing and destressing processes. With HPHT treatment, the EBSD map reveals a uniform distribution of the B2 phase, without any significant phase segregation. Thus, the plastic deformation ability of the B2 phase can be tuned.

Although the polygon-like structure in Ac+HPHT alloy is not as regular as the hexagonal structure in the Ho+HPHT alloy, the specific structure caused by HPHT can also provide greater opportunity for achieving enhanced mechanical properties and help to arrest cracking along the interfaces. As shown in Supplementary Fig. 11k, the Ac+HPHT alloy also shows large differences in crack morphology with the Ac alloy (Supplementary Fig. 11c). The elongated hollows with large widths along interfaces appeared in the fractured Ac alloy, which indicates that boundary-induced cracks dominate the eventual fracture process of the Ac alloy. By contrast, we did not observe the long crack in the fractured Ac+HPHT alloy surface. The micrograph of the fractured Ac+HPHT alloy shows a high density of microcracks on the surface, indicating an enhanced damage resistance of the Ac+HPHT alloy. The microcracks can cut through the FCC and B2 phases in the Ac+HPHT alloy, indicating that high local stresses were shielded and plastic

deformation was better accommodated to dissipate the strain energy[11]. Also, the B2 phase with high stability can isolate the microcracks and prevent microcracks coalescing into elongated voids. Furthermore, it is noted that these two phases can deflect crack paths, thereby delaying the crack extension and hence suppressing its cracking. These discrepancies are derived from the significant differences in plastic deformation behavior. Owing to the weak interface between the FCC and B2 phase in the Ac alloy, interfacial cavitation starts to occur upon deformation leading to the growth of large cracks. Due to the specific architecture in the Ac+HPHT alloy, more profuse plastic activities occur in the soft FCC phase and especially the hard B2 phase. Thus, smaller cracks are generally observed in the Ac+HPHT alloy than those in the Ac alloy. Overall, the polygon-like structure in the Ac+HPHT alloy contributes to enhanced crack resistance and improved plasticity. The comparison between the Ac+HPHT and Ac alloys further confirms the ability of our HPHT technique.

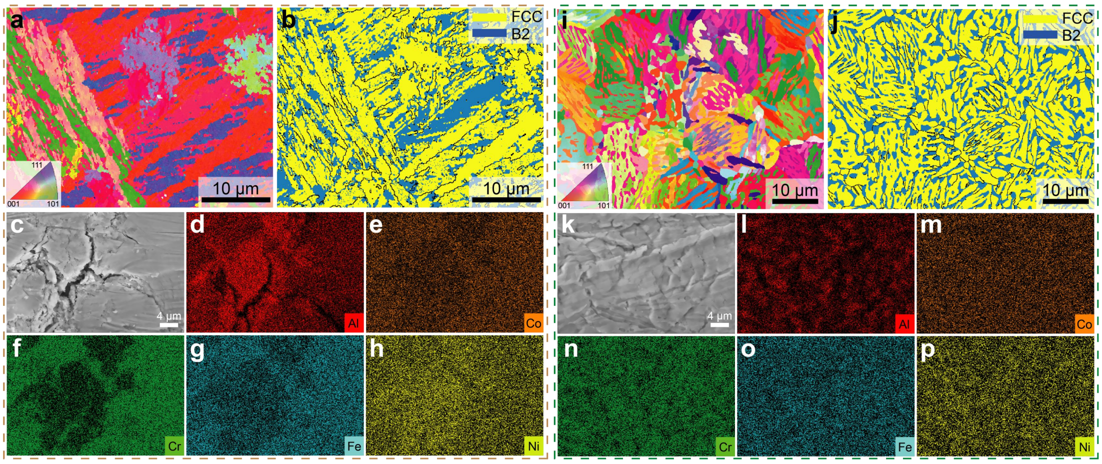

Supplementary Fig. 11 | The microstructure and crack behavior of the Ac and Ac+HPHT $Al_{0.7}CoCrFeNi$ alloys. a, An electron backscatter diffraction (EBSD) inverse-pole figure (IPF) map of the Ac $Al_{0.7}CoCrFeNi$ alloy. **b,** The corresponding EBSD phase map reveals that the dual-phase lamellae consist of alternating FCC layers and BCC layers in the Ac alloy. **c,** SEM secondary electron (SE) image of the fracture surface in the Ac alloy. **d-h,** The corresponding element mapping for the enlarged image of the fracture surface in the Ac alloy. **i,** The electron backscatter diffraction (EBSD) inverse-pole figure (IPF) map of the Ac+HPHT $Al_{0.7}CoCrFeNi$ alloy. **j,** The corresponding EBSD phase map reveals that the dual-phase lamellae structure has been modified in the Ac+HPHT alloy. **k,** SEM secondary electron (SE) image of the fracture surface in the Ac+HPHT alloy. **l-p,** The corresponding element mapping for the enlarged image of the fracture surface in the Ac+HPHT alloy.

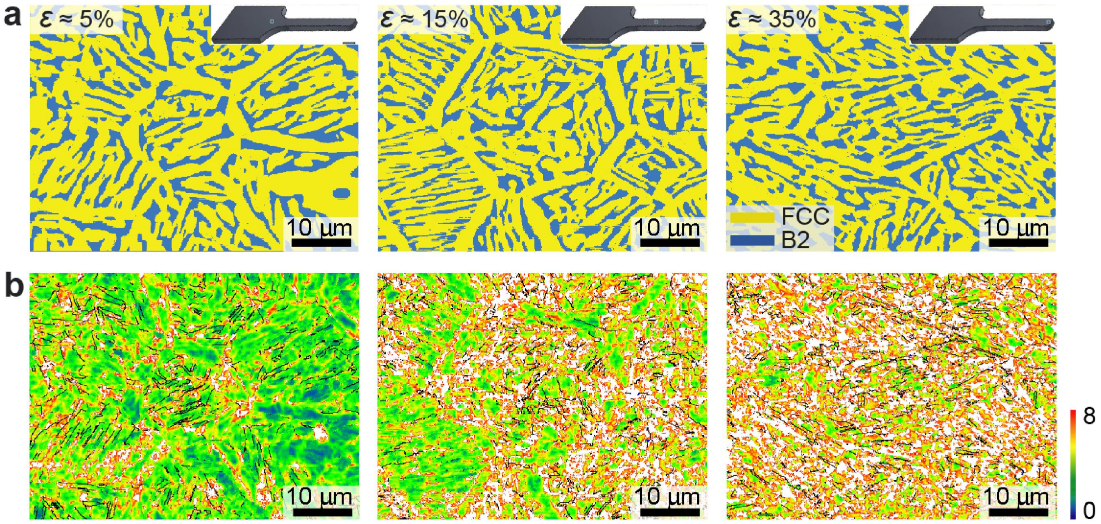

Supplementary Fig. 12 | Microstructure evolution of the HPHT-treated alloy at different strain amplitudes. a, Electron backscatter diffraction (EBSD) phase distribution maps at the strain amplitudes of 5%, 15%, and 35%. The regions at different strain amplitudes for EBSD analysis of the deformed HPHT-treated alloy are shown in the inset. The scale bar is 0.35 mm. **b,** The corresponding geometrically necessary dislocations (GND) maps. The color bar in GND maps indicates the range of GND density from 0 to 8×10^{14} m^{-2}.

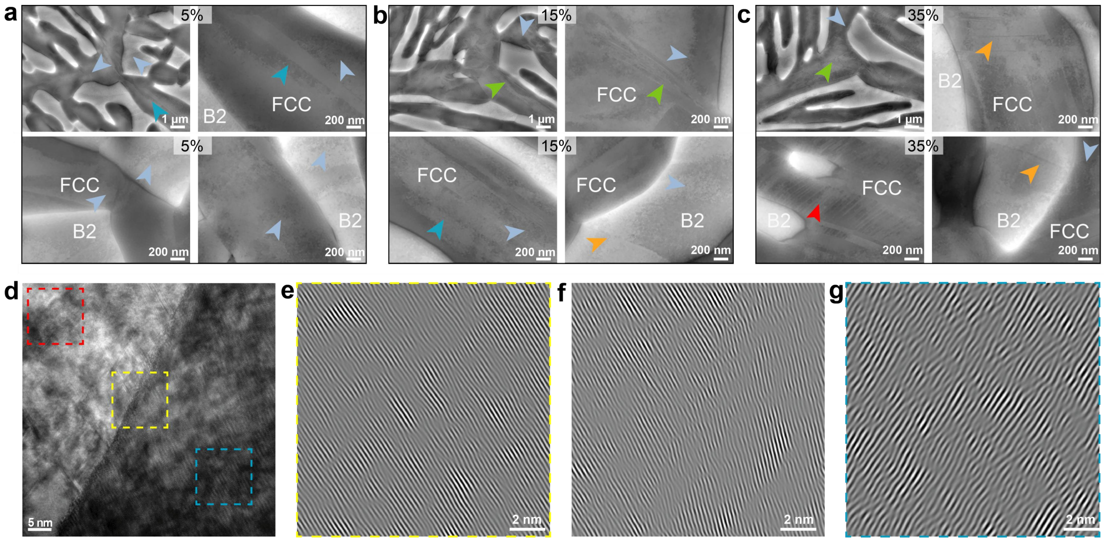

Supplementary Fig. 13 | Deformation structures in the HPHT-treated alloy at different strain amplitudes. a-c, Electron contrast channel imaging (ECCI) analyses reveal the evolution of the deformation substructure in the HPHT-treated alloy at the strain amplitudes of 5%, 15%, and 35%. **d,** HRTEM image shows tthe FCC phase (the region highlighted by the red dashed box), interface (the region highlighted by the yellow dashed box) and BCC phase (the region highlighted by the blue dashed box) in the Ho alloy. **e-g,** The corresponding IFFT images of the FCC phase (the region highlighted by the red dashed box in k), interface (the region highlighted by the yellow dashed box in k) and BCC phase (the region highlighted by the blue dashed box in k).

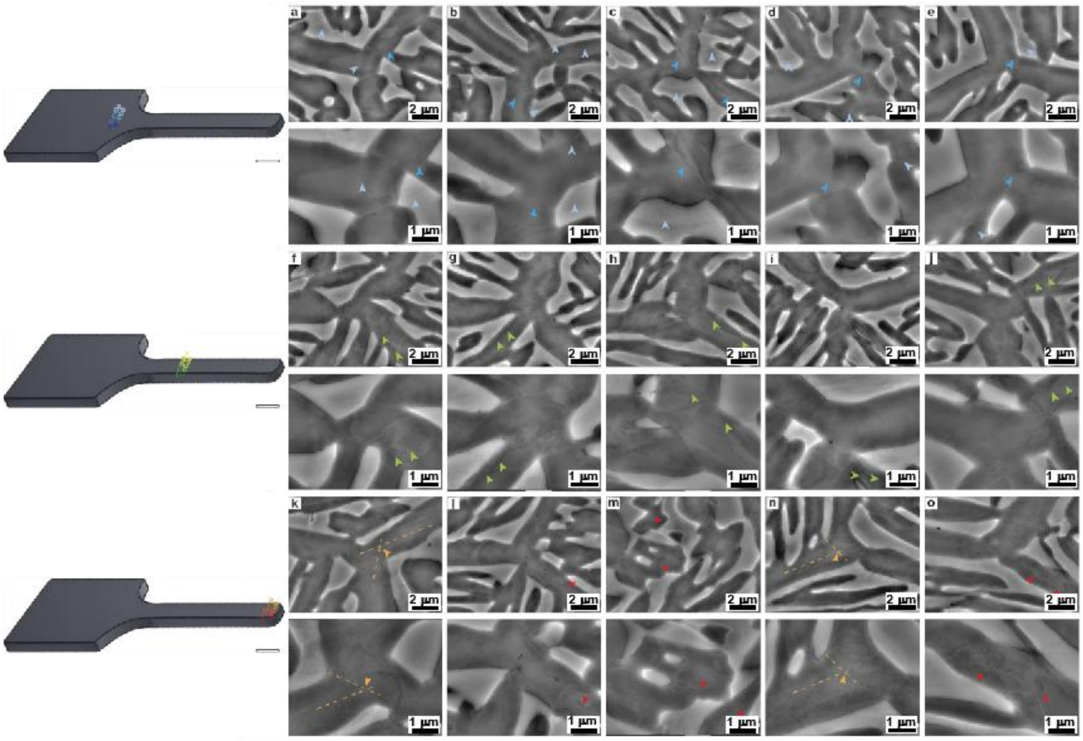

Supplementary Fig. 14 | Electron contrast channel imaging (ECCI) analyses reveal the evolution of the deformation substructure in the HPHT-treated alloy at different strain amplitudes. Five different locations of the fractured HPHT-treated alloy (scale bar is 0.35 mm) were measured at the same strain amplitude. **a-e,** 5%. **f-j,** 15%. **k-o,** 35%.

Supplementary Note 4 | Microstructure evolution in the Ho alloy at different strain amplitudes

The microstructure evolution in the tensile-deformed Ho alloy at various strain amplitudes was analyzed by EBSD (Supplementary Fig. 15). The corresponding EBSD phase maps confirm that the dual-phase structure is maintained during tension without deformation-induced phase transformation (Supplementary Fig. 15b). The distribution of plastic deformation in the Ho alloy at different strain amplitudes can be analyzed using the kernel average misorientation (KAM) maps[12] (Supplementary Fig. 15c). At small plastic strains, the FCC phase exhibits relatively higher KAM value than the B2 phase. With increasing strain, the FCC phase possesses higher KAM values and the BCC phase exhibits low KAM, which results in strain localization. In particular, a relatively inhomogeneous distribution of KAM is observed at the final fracture with a plastic strain of 15%, which results in strain localization and contributes to the limited tensile elongation of the Ho alloy.

To quantify the plastic deformation accommodation in FCC and BCC phases, we have calculated the average densities of geometrically necessary dislocations (GNDs)[13,14] under various strains for the Ho alloy (Supplementary Fig. 15d). From 0% to 3% strains, the GND density of the FCC phase increases rapidly from 3.52×10^{14} m^{-2} to 6.53×10^{14} m^{-2}, whereas the GND density of the B2 phase increases from 3.09×10^{14} m^{-2} to 4.81×10^{14} m^{-2}. Upon further loading, the GND density of the FCC phase increases much faster than that of the B2 phase, eventually resulting in a severe mismatch of the average GND density between the FCC and B2 phase phases, consistent with the highly incompatible plastic strain distribution in the Ho alloy. The GND maps in the Ho alloy at different strain amplitudes are shown in Supplementary Fig. 16. It can be seen that the B2 phase possesses much smaller GND values than the FCC phase in the later stage of tensile deformation. These observations suggest a limited capability of dislocation storage in the Ho alloy.

To further reveal the underlying deformation mechanisms, we performed electron contrast channel imaging (ECCI) analysis at various strains for the Ho alloy (Supplementary Figs. 17a-c). We have carried out five individual ECCI analyses for different locations at the same strain amplitude to confirm the observed deformation characteristics of the HPHT-treated alloy (Supplementary Fig. 18). The corresponding magnified ECCI images are presented in Supplementary Figs. 17d-f. At a strain of 3%, few dislocations were observed in the FCC phase

and no dislocations in the B2 phase. With strain increasing to 7%, stacking faults (SFs) were activated in the FCC phase, indicating enhanced dislocation activities in the FCC phase. Extensive dislocation pile-up was observed around the B2 phase and almost no dislocation in the B2 phases, suggesting no plastic deformation occurred in the B2 phases. At 15% strain, the limited slip lines only exist in the FCC phase while the BCC phase exhibits no evident dislocation activities. Dislocation pile-ups were evident around the fine B2 phase. Note that there was no evidence of HDDWs and DTs in the Ho alloy at fracture strain. The microstructure evolution intrinsically indicates relatively low dislocation accumulation in the Ho alloy, in which the internal stress is insufficient to activate the HDDWs and DTs activity[15-17].

TEM imaging of the fracture region also demonstrates localized deformation at the interface and the absence of dislocations in the B2 phase in the Ho alloy. Supplementary Figs. 17g-i display the microstructure of the Ho alloy captured at 15% strain. A large number of dislocations appeared in the fcc phase, while no obvious dislocations were observed in the B2 phase. The dislocation slip was blocked in the fcc/B2 phase boundary. A higher dislocation density was seen in the fcc phase near the fcc/B2 phase boundary, compared to the dislocation density further inside the fcc phase. Except for the stacking faults (Supplementary Fig. 17j), no detectable twinning was observed under TEM. The HRTEM image near the FCC/B2 phase interface and the corresponding inverse fast Fourier transformation (FFT) patterns of the FCC, interface and BCC are presented in Supplementary Figs. 17k-n. A transition layer between the FCC and B2 phases can be seen. More edge dislocations were observed in the FCC phase and interface region, while almost none can be seen in the B2 phase. This observation provides evidence of dislocation accumulation at the phase interfaces in the Ho alloy, which has been frequently reported in eutectic high entropy alloys and widely regarded as the origin of interface-induced fracture[18-23].

These results indicate that the localized deformation of the Ho alloy is strongly related to the deformation incompatibility of the FCC and B2 phases. The microstructure evolution of the Ho alloy has been summarized in Supplementary Fig. 19. During deformation in the Ho alloy, mobile dislocations are generated and slip in the soft phases first, and pile-ups will occur near phase boundaries. With increasing applied stress, the continuous accumulation of dislocations will lead to stress concentration. The dislocation pile-ups do not lead to deformation in hard B2 phases, but they do cause crack formation between FCC and B2 phases. The lack of deformability in the B2

phase makes the Ho alloy unable to attain global plasticity. The strong strain incompatibility between different phases and the limited dislocation storage ability lead to the initiation and propagation of microcracks along the relatively weak interfaces. In this respect, what HPHT achieves is to modify the structure to promote synchronous deformation of the FCC and B2 phases.

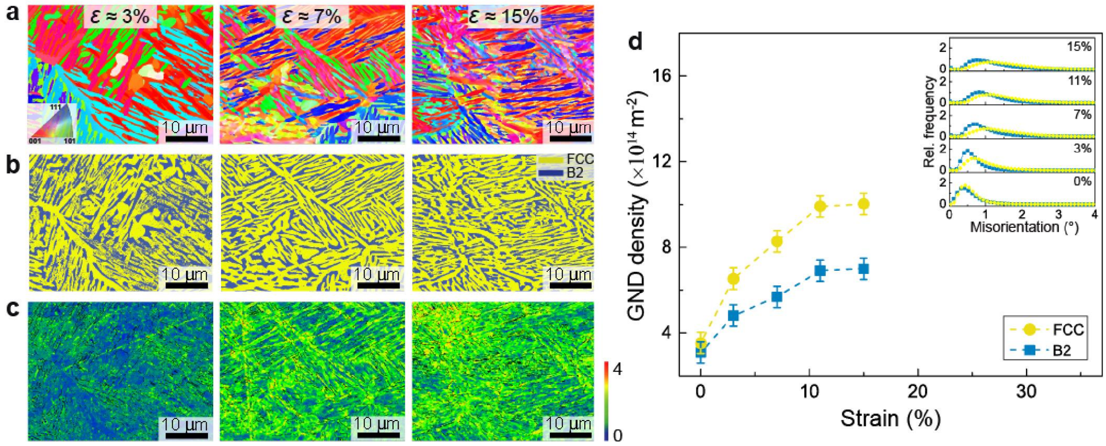

Supplementary Fig. 15 | The microstructure evolution of the Ho alloy at different strain amplitudes. a, Electron backscatter diffraction (EBSD) inverse-pole figure (IPF) maps at the strain amplitudes of 3%, 7%, and 15%. **b,** The corresponding phase distribution maps. **c,** The corresponding kernel average misorientation (KAM) distribution maps. **d,** Variations of the average geometrically necessary dislocations (GND) density in the FCC and B2 phases, respectively. The error bars represent the corresponding standard deviation, which are obtained from 3 independent EBSD mappings on the regions with identical local strain levels. The inset shows the variation in the average misorientation of the Ho alloy with increasing plastic strain.

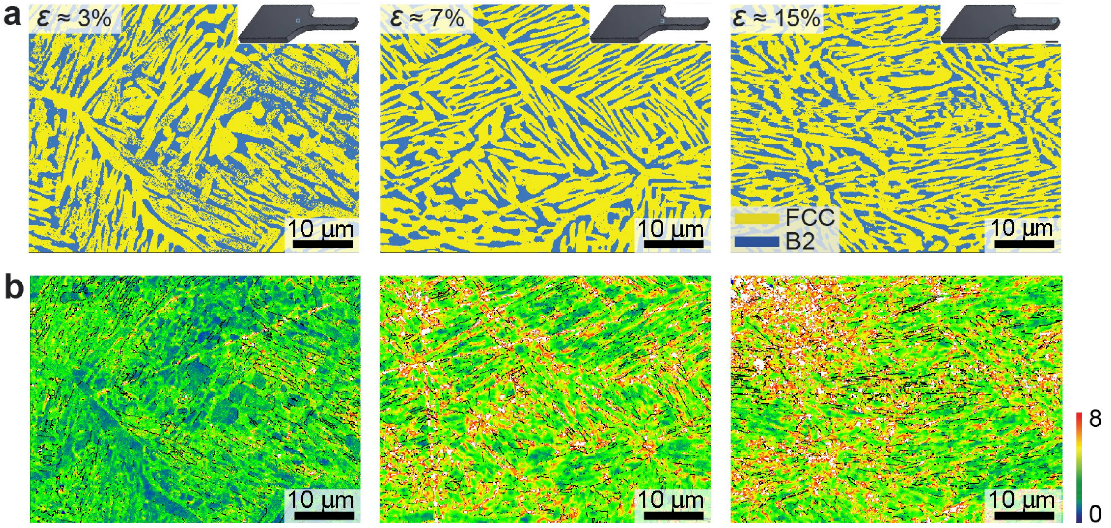

Supplementary Fig. 16 | Microstructure evolution of the Ho alloy at different strain amplitudes. a, Electron backscatter diffraction (EBSD) phase distribution maps at the strain amplitudes of 3%, 7%, and 15%. The regions at different strain amplitudes for EBSD analysis of the deformed Ho alloy are shown in the inset. The scale bar is 0.35 mm. **b,** The corresponding geometrically necessary dislocations (GND) maps. The color bar in GND maps indicates the range of GND density from 0 to 8×10^{14} m^{-2}.

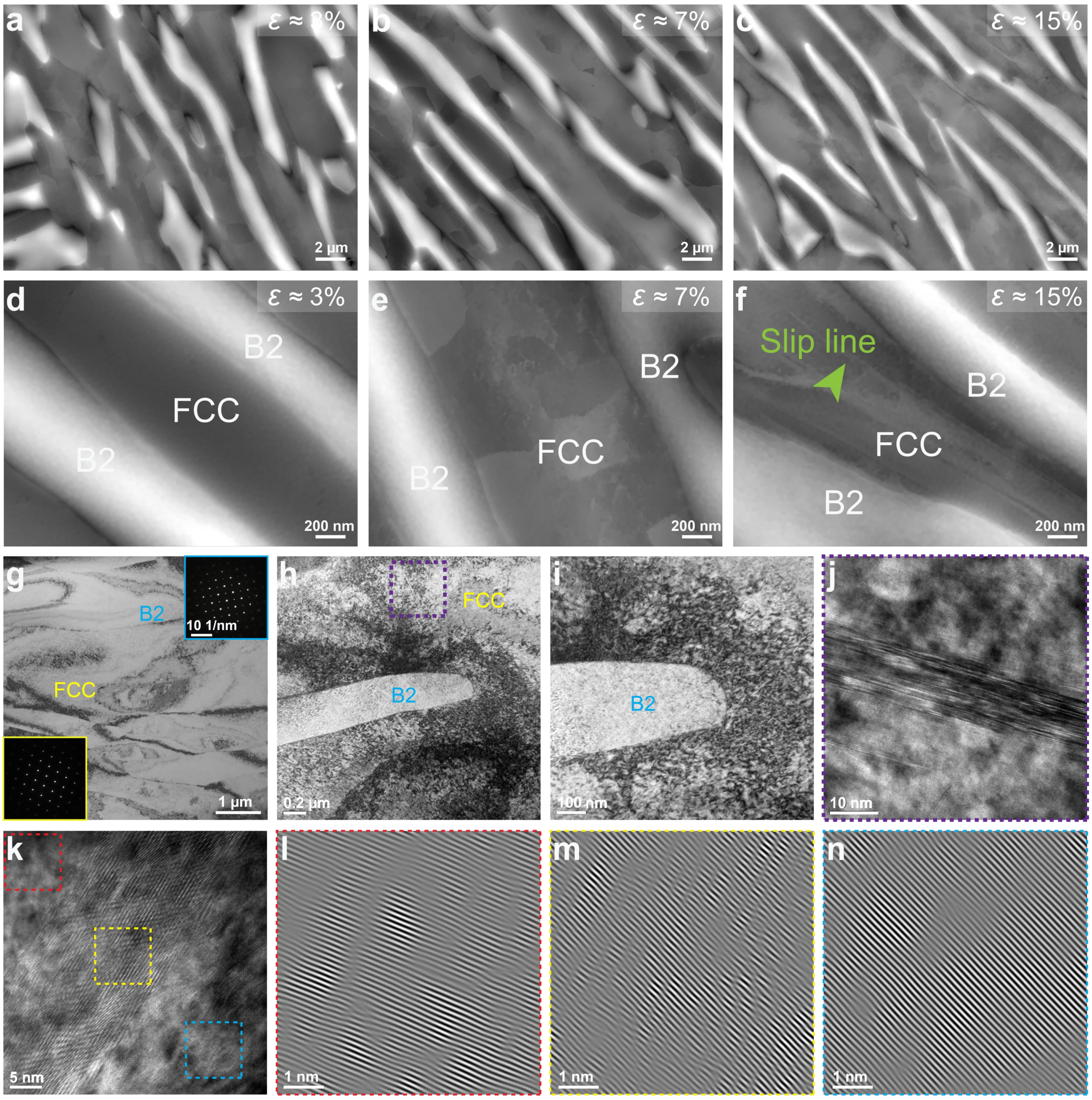

Supplementary Fig. 17 | Deformation substructures in the Ho alloy at different strain amplitudes. a-f, ECCI images reveal the evolution of the deformation substructure in the Ho alloy at strain amplitudes of 3%, 7%, and 15%. **g,** TEM image of the plastically deformed Ho alloy at 15% tensile strain, showing some obvious dislocations in the FCC phases. The insets show the related selected area electron diffraction (SAED) patterns of FCC and B2 phases (indicated by yellow and blue colors, respectively). **h** and **i,** HRTEM micrographs of the deformation substructures at 15% tensile strain. **j,** Deformation-induced stacking faults in the FCC phase. **k,** HRTEM image showing the FCC phase (the region highlighted by the red dashed box), interface (the region highlighted by the yellow dashed box) and BCC phase (the region highlighted by the blue dashed box) in the Ho alloy. **l-n,** The corresponding IFFT images of the FCC phase (the region highlighted by the red dashed box in k), interface (the region highlighted by the yellow dashed box in k) and BCC phase (the region highlighted by the blue dashed box in k).

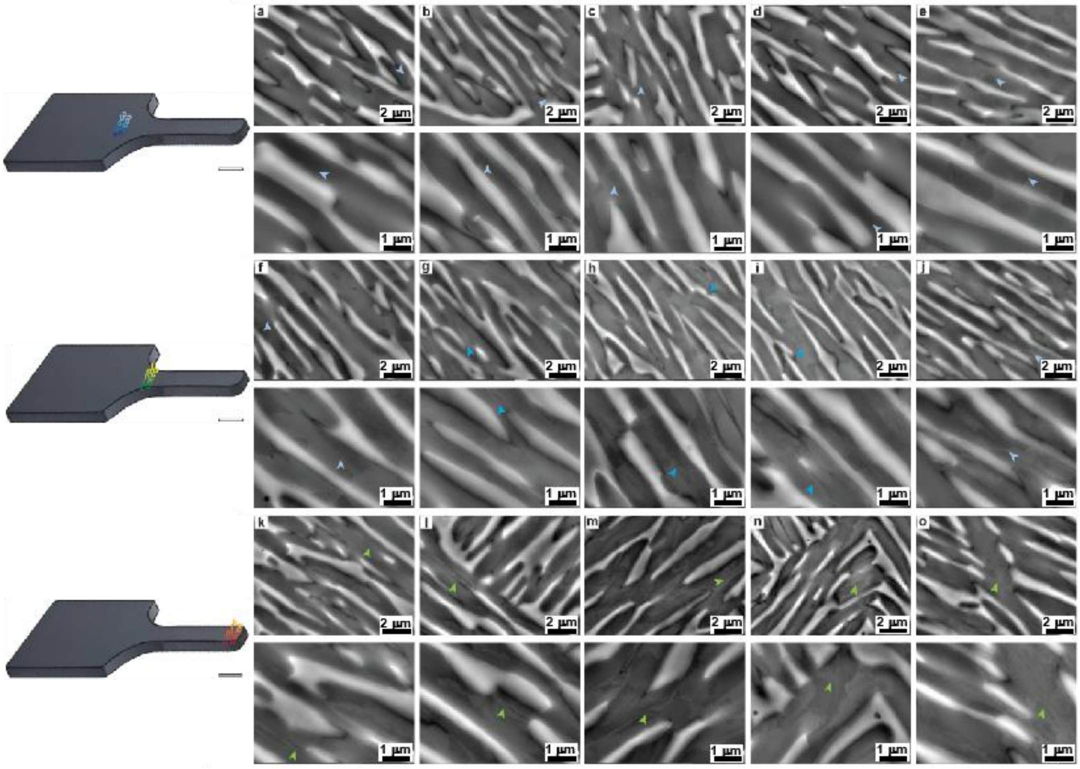

Supplementary Fig. 18 | Electron contrast channel imaging (ECCI) analyses reveal the evolution of the deformation substructure in the Ho alloy at different strain amplitudes. Five different locations of the fractured Ho alloy (scale bar is 0.35 mm) were measured at the same strain amplitude. **a-e,** 3%. **f-j,** 7%. **k-o,** 15%.

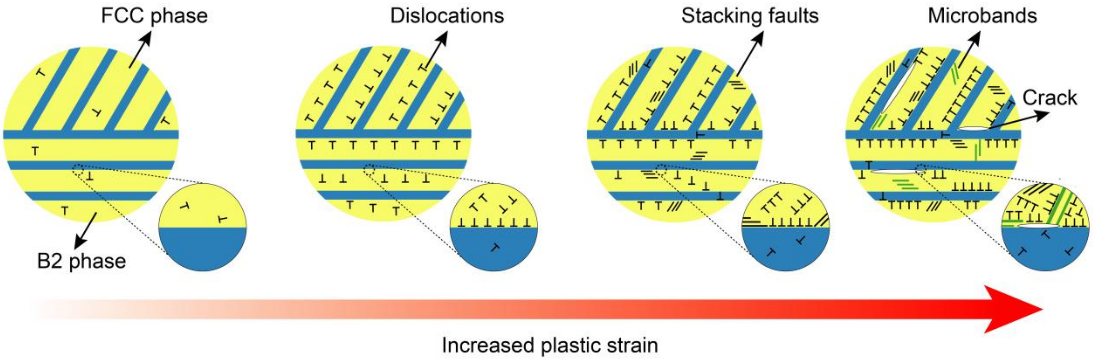

Supplementary Fig. 19 | Schematic sketch of microstructure evolution with increased plastic strain in the Ho alloy. The Ho alloy with a lamellae microstructure primarily deforms by the mechanism of the dislocation pile-up. Due to the highly incompatible plastic strain distribution, only limited stacking faults and microbands exist in the FCC phase while the BCC phase exhibits no evident dislocation activities. The local stress concentration caused by the accumulation of dislocations at the interface (shown in the enlarged sketch maps) leads to the onset of micro-cracking.

Supplementary Note 5 | The evolution of total dislocation density in HPHT-treated and Ho alloys

The total dislocation density can be estimated from the X-ray diffraction (XRD) patterns by employing the Williamson-Hall method[24]. The XRD patterns of the tensile-deformed Ho and HPHT-treated alloy at various strain amplitudes are presented in Supplementary Fig. 20.

In this approach, after instrument broadening is deducted from the observed peak broadening, the true XRD peak broadening β consists of the crystallite size broadening β_G and the strain broadening β_S, that is:

$$\beta = \beta_G + \beta_S \tag{1}$$

$$\beta_G = K\lambda / (D * \cos\theta) \tag{2}$$

$$\beta_S = 4\varepsilon * \tan\theta \tag{3}$$

where β is the fullwidth at half maximum (FWHM) of the main peak, K is a constant (~ 0.9), λ is the wavelength of Co K_α radiation, D is the crystallite size, ε is the microstrain and θ is the Bragg angle of the certain peak. By substituting equations (2) and (3) in equation (1), equation (1) can be rewritten as

$$\beta_{\mathrm{hkl}}\cos\theta_{\mathrm{hkl}} = K\lambda / D + (4\sin\theta_{\mathrm{hkl}}) * \varepsilon \tag{4}$$

As seen in equation (4), the ε is the slope of the linear fit of the $\beta_{\mathrm{hkl}}\cos\theta_{\mathrm{hkl}} - 4\sin\theta_{\mathrm{hkl}}$ plot; the size of the crystallites D can be obtained from the intercept. Then, the dislocation density ρ can be calculated by the following equation:

$$\rho = 2\sqrt{3} * \varepsilon / (D\mathrm{b}) \tag{5}$$

where ε is the average microstrain, D is the crystallite size, b is the modulus of Burgers vector.

The calculated dislocation densities are illustrated in Supplementary Fig. 21. As shown in Supplementary Fig. 21a, ρ_{FCC} of the Ho alloy increases from 2.68×10^{14} m^{-2} to 3.75×10^{14} m^{-2} from 0% to 3% strains, whereas the ρ_{B2} increases from 7.51×10^{13} m^{-2} to 1.81×10^{14} m^{-2}. Upon further loading, the dislocation density of the FCC phase increases to 9.79×10^{14} m^{-2} at 15% strain,

which is higher than that of the B2 phase. In the HPHT-treated alloy (Supplementary Fig. 21b), the dislocation density of the FCC phase increases rapidly from 2.86×10^{14} m^{-2} to 6.99×10^{14} m^{-2} from 0% to 15% strains, whereas the dislocation density of the B2 phase increases from 1.03×10^{14} m^{-2} to 1.04×10^{15} m^{-2}. Upon further loading, the dislocation density of the B2 phase increases to 1.91×10^{15} m^{-2} at 35% strain, whereas the dislocation density of the FCC phase increases to 1.63×10^{15} m^{-2} at 35% strain. These results confirm the uniform deformation of the HPHT-treated alloy. In addition, the dislocation densities in the HPHT alloy are much higher than those in the Ho alloy, again suggesting the improved capability of dislocation storage in the HPHT-treated alloy.

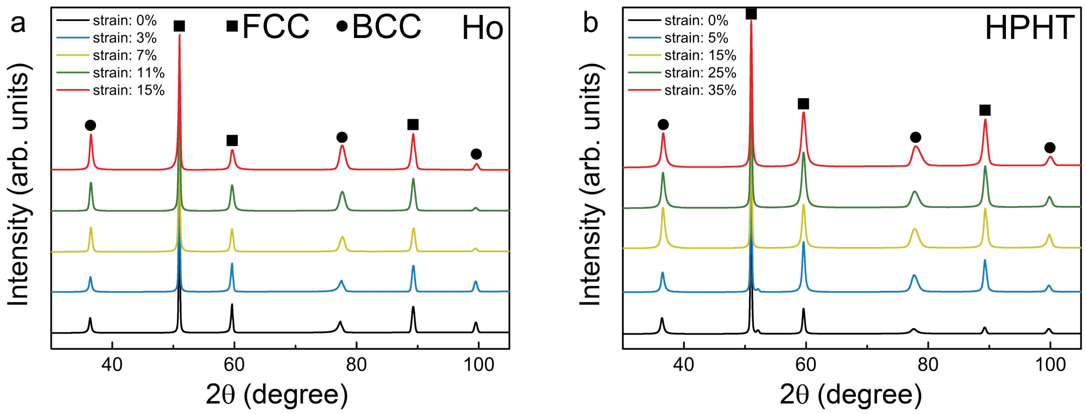

Supplementary Fig. 20 | The X-ray diffraction patterns of the Ho and HPHT-treated alloys at different strain amplitudes.

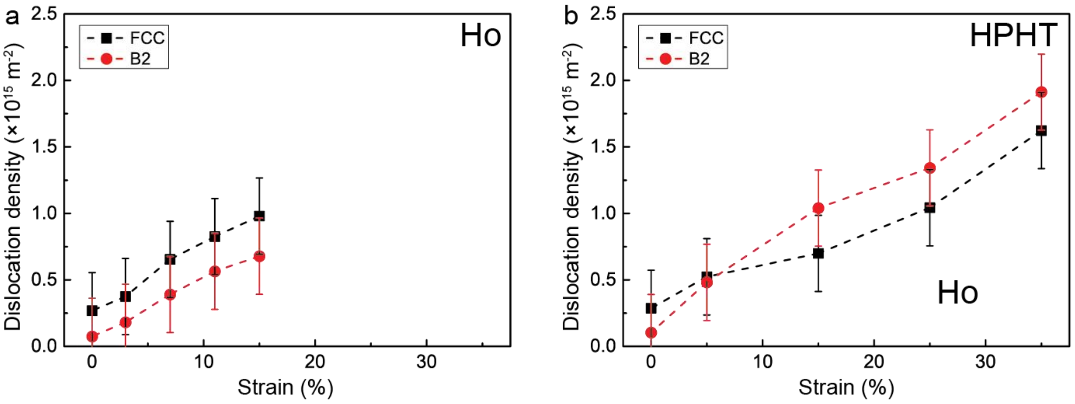

Supplementary Fig. 21 | Total dislocation density evolution at different strain amplitudes. a, Dislocation density against strain in the FCC and the B2 phases for the Ho alloy. Error bars represent the standard deviation. **b,** Dislocation density against strain in the FCC and the B2 phases for the HPHT-treated alloy. Error bars represent the standard deviation.

Supplementary Note 6 | The effect of HPHT treatment on the microstructure and mechanical properties of pure copper and other alloys

We demonstrate that the HPHT treatment can enhance the strength and ductility of not only the eutectic high entropy alloy under study but also other metallic material systems, even simple metals like copper (Cu). To this purpose, the pure Cu specimen was homogenized at 1073 K for 6h and then water-quenched to ambient temperature. The obtained sample is referred to as the Cu-Ho sample. To study the effect of HPHT treatment on the mechanical properties of pure Cu, the Cu-Ho sample was treated at 6 GPa and 1273 K for 2 h, which is denoted as Cu-HPHT. The EBSD phase maps of the Cu-Ho and Cu-HPHT samples reveal that the structure of Cu-Ho can be engineered by HPHT treatment. As shown in Supplementary Fig. 22, the Cu-HPHT sample exhibits a significant reduction in grain size and a uniform distribution of grains compared to the Cu-Ho sample. In addition to grain refinement, the HPHT treatment also introduced more grain boundaries. It is known that grain refinement is an effective way to evade grain boundary embrittlement because more interfaces reduce the level of individual GB segregation[25-27]. TEM technique was used to better characterize the microstructures of the Cu-Ho (Supplementary Fig. 22a) and Cu-HPHT (Supplementary Fig. 22e) samples. The corresponding selected area electron diffraction (SAED) patterns of the grains suggest that only the FCC phase exists in the Cu-Ho sample (Supplementary Fig. 22b). The high-resolution TEM image (Supplementary Fig. 22c) reveals a grain boundary structure with a transition layer between neighbouring grains in the Cu-Ho sample, which is consistent with previous work[28,29]. The corresponding inverse FFT image (Supplementary Fig. 22d) shows that the boundary contains a large amount of misfit dislocations. Compared to the Cu-Ho sample, the grain boundary seems to become smoother in the Cu-HPHT sample, as shown in Supplementary Fig. 22f. The high-resolution TEM image (Supplementary Fig. 22g) reveals an extremely sharp grain boundary in the Cu-HPHT sample. The corresponding selected area electron diffraction (SAED) pattern shows that the two grains have a specific orientation relationship of $(011)//(01\bar{1})$, which meets the classical Kurdjumov-Sachs (KS) relationship[30]. Further analysis shows that the two grains possess a mismatch factor of merely 0.002, indicating that the grain boundary has high cohesion in the Cu-HPHT sample. The corresponding IFFT image (Supplementary Fig. 22h) confirms the high degree of lattice matching

between the two grains. These results demonstrate that the HPHT treatment can result in the enhanced cohesion of grain boundaries in pure copper. Presumably, the formation of such grain boundary is energetically favorable[31] and may be beneficial to improve the mechanical properties.

Supplementary Fig. 22i displays the tensile properties of the Cu-Ho and Cu-HPHT samples at room temperature. The HPHT-treated sample exhibits greatly improved fracture strength and tensile ductility compared with those of the Ho sample. It is known that grain refinement can render material stronger, but this process is usually accompanied by a dramatic loss of ductility[32-36]. However, the Cu-HPHT sample with reduced grain size achieves enhanced properties with a strength-ductility synergy, although the ductility does not increase as much as strength. The HPHT-treated Cu alloy actually exhibits an exceptional combination of high strength and large uniform elongation, in comparison with the enhanced mechanical properties observed in the well-known gradient nano-grained Cu sample[37]. The improved mechanical properties of the Cu-HPHT sample arise from the enhanced grain boundary cohesion and underlying plastic accommodation behavior. A relatively homogeneous distribution of KAM is observed in the Cu-HPHT sample at the final fracture, contributing to its extra tensile elongation. In contrast, the grains possess different KAM values in the Cu-Ho sample, indicating highly incompatible plastic strains.

To verify that our HPHT strategy can also be implemented for other metallic materials, we have performed the same heat treatment engineering protocol for the $Fe_{50}Mn_{27}Ni_{10}Cr_{13}$ alloy. Supplementary Fig. 23 displays the tensile properties of the Ho and HPHT-treated $Fe_{50}Mn_{27}Ni_{10}Cr_{13}$ samples at room temperature. The HPHT-treated sample exhibits greatly improved fracture strength compared with those of the Ho sample. Usually, increasing the yield strength and fracture strength of a material requires sacrificing its ductility. However, we achieved an increase in fracture strength without compromising the plasticity of the material. The TEM technique was used to better characterize the microstructures of the Ho $Fe_{50}Mn_{27}Ni_{10}Cr_{13}$ alloy and HPHT-treated Fe-based alloy samples. The corresponding selected area electron diffraction (SAED) patterns of the grains suggest that only the FCC phase exists in the Ho $Fe_{50}Mn_{27}Ni_{10}Cr_{13}$ sample (Supplementary Fig. 23b). The high-resolution TEM image reveals a grain boundary structure with a transition layer between neighboring grains in the Ho $Fe_{50}Mn_{27}Ni_{10}Cr_{13}$ sample (Supplementary Fig. 23c). Compared to the Ho $Fe_{50}Mn_{27}Ni_{10}Cr_{13}$ sample, the grain boundary

seems to become smoother in the HPHT-treated $Fe_{50}Mn_{27}Ni_{10}Cr_{13}$ sample, as shown in Supplementary Fig. 23d. The high-resolution TEM image (Supplementary Fig. 23e) reveals an extremely sharp grain boundary in the HPHT-treated $Fe_{50}Mn_{27}Ni_{10}Cr_{13}$ sample. The corresponding selected area electron diffraction (SAED) pattern shows that the two grains have a specific orientation relationship of $(11\bar{1})//(200)$, which meets the classical Kurdjumov-Sachs (KS) relationship[30]. The formation of such grain boundary is energetically favorable and may be beneficial to improve the mechanical properties.

In addition, we have also performed additional experiments using the HPHT treatment engineering protocol for the $AlCoCrFeNi_{2.1}$ alloy. As shown in Supplementary Fig. 24, the HPHT-treated $AlCoCrFeNi_{2.1}$ alloy exhibits improved fracture strength and ductility compared with those of the Ho $AlCoCrFeNi_{2.1}$ alloy. In addition, the dual-phase lamellae structure frequently observed in the Ho alloy can be transformed into a hierarchically patterned microstructure. Although the effect of HPHT treatment is related to alloy composition, initial atomic structure and processing conditions, we have clearly demonstrated that the HPHT treatment can help overcome the strength-ductility trade-off in various eutectic HEAs.

These results thus provide strong evidence that the HPHT strategy proposed in the present work is not only limited to eutectic high entropy alloys or pure copper, but is promising to be implemented for other alloy systems. These findings suggest a promising general paradigm for achieving strength-ductility synergy by engineering the microstructure of metals and alloys through HPHT. The simplicity of this processing route can be attractive for many industrial applications. Meanwhile, we anticipate that the HPHT strategy can be even further extended to non-metallic systems or other physical properties. Our findings are calling for future work on comprehensive studies of the discovery of novel high-performance HPHT-treated alloys.

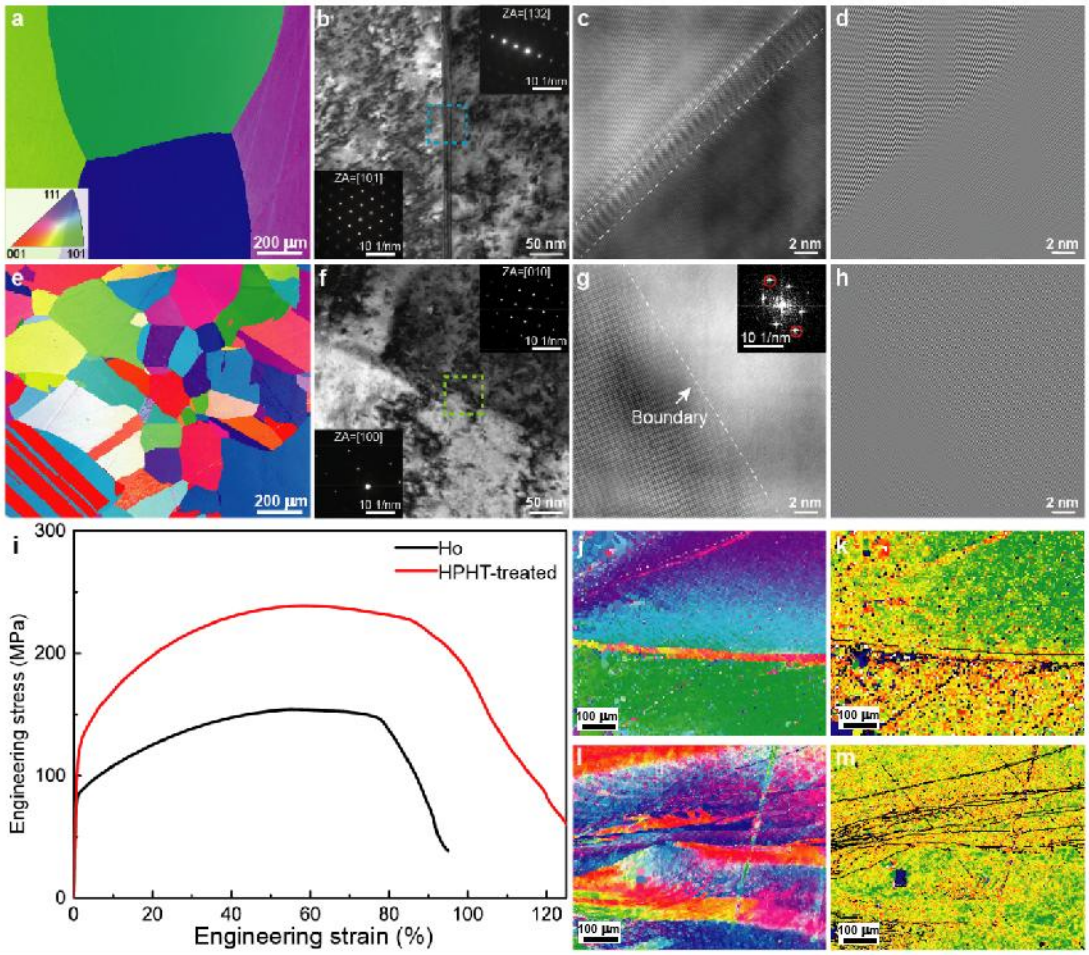

Supplementary Fig. 22 | The effect of HPHT treatment on the microstructure and mechanical properties of pure copper (Cu). a, The electron backscatter diffraction (EBSD) inverse-pole figure (IPF) map of the Cu-Ho sample, which serves as a precursor material for further HPHT treatment. **b,** Low magnification TEM image of the Cu-Ho sample, showing a transition layer at the interface. The insets show related SAED patterns of the grains. **c** and **d,** Typical HRTEM image and the corresponding IFFT image of the Cu-Ho sample, which demonstrates the incoherent interface. **e,** The EBSD-IPF map of the Cu-HPHT sample. **f,** Low magnification TEM image of the Cu-HPHT sample. The insets show related SAED patterns of the interface region, which meets the K-S relationship. **g** and **h,** Typical HRTEM image and the corresponding IFFT image of the Cu-HPHT sample, which demonstrates a coherent interface. **i,** Engineering stress-strain curves of the Cu-HPHT sample compared to that of the Cu-Ho sample, showing a synergistic enhancement of both strength and ductility. **j** and **k,** The electron backscatter diffraction (EBSD) inverse-pole figure (IPF) map and corresponding KAM distribution maps of the Cu-Ho sample at the fracture strain. **l** and **m,** The electron backscatter diffraction (EBSD) inverse-pole figure (IPF) map and the corresponding KAM distribution maps of the Cu-HPHT sample at the fracture strain.

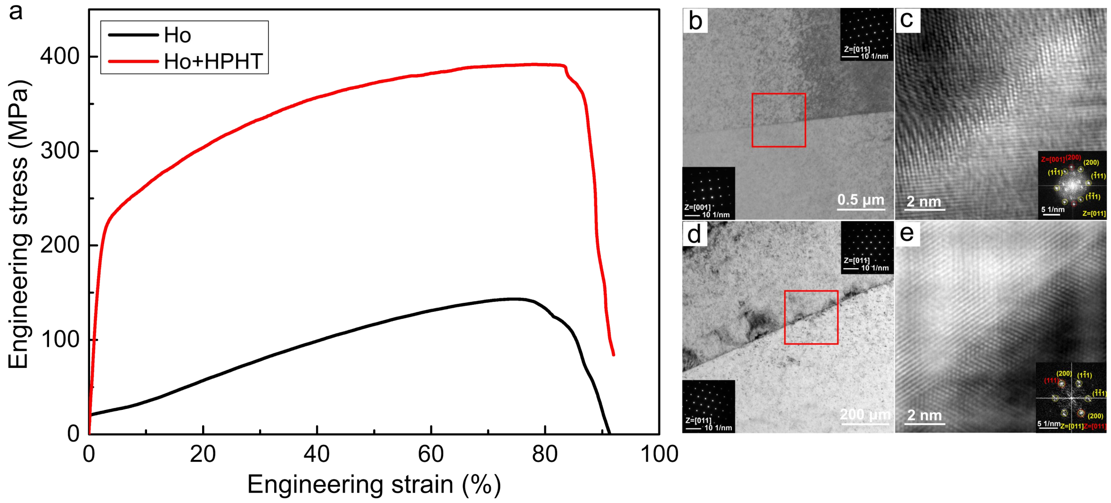

Supplementary Fig. 23 | The effect of HPHT treatment on the microstructure and mechanical properties of $Fe_{50}Mn_{27}Ni_{10}Cr_{13}$ alloy. a, Engineering stress-strain curve of the HPHT-treated $Fe_{50}Mn_{27}Ni_{10}Cr_{13}$ sample, in comparison with that of the Ho $Fe_{50}Mn_{27}Ni_{10}Cr_{13}$ sample, showing a synergistic enhancement in both strength and ductility. **b,** Low magnification TEM image of the Ho $Fe_{50}Mn_{27}Ni_{10}Cr_{13}$ sample, showing a transition layer at the interface. The insets show related SAED patterns of the grains. **c,** Typical HRTEM image of the Ho $Fe_{50}Mn_{27}Ni_{10}Cr_{13}$ sample, which demonstrates the incoherent interface. **d,** Low magnification TEM image of the HPHT-treated $Fe_{50}Mn_{27}Ni_{10}Cr_{13}$ sample. The insets show related SAED patterns of the interface region, which meets the K-S relationship. **e,** Typical HRTEM image and the corresponding IFFT image of the HPHT-treated $Fe_{50}Mn_{27}Ni_{10}Cr_{13}$ sample, which demonstrates a coherent interface.

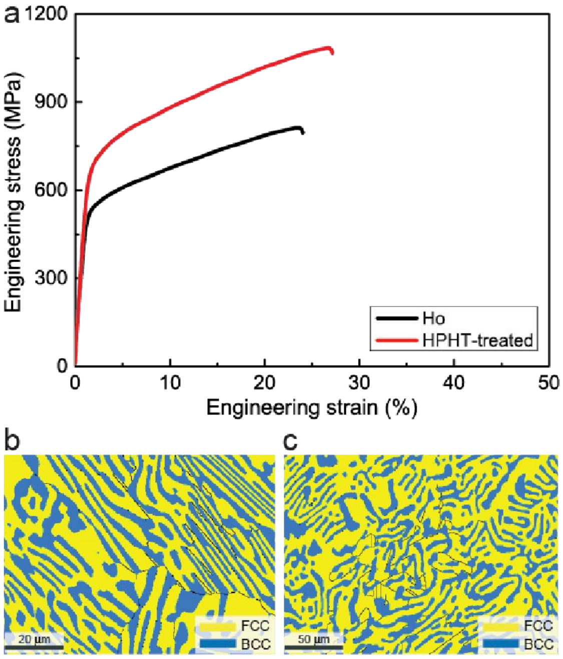

Supplementary Fig. 24 | The effect of HPHT treatment on the microstructure and mechanical properties of $AlCoCrFeNi_{2.1}$ alloy. a, Engineering stress-strain curves of HPHT-treated $AlCoCrFeNi_{2.1}$ sample and Ho $AlCoCrFeNi_{2.1}$ sample. **b,** The corresponding EBSD phase map reveals the dual-phase lamellae composed of alternating FCC layers and BCC layers in the Ho alloy. **c,** The corresponding EBSD phase maps reveal the modified dual-phase lamellae structure in the HPHT-treated alloy.

References

1. Bocanegra-bernal, M. H. Hot Isostatic Pressing (HIP) technology and its applications to metals and ceramics. *J. Mater. Sci.* **39**, 6399-6420 (2004).
2. Yang, R. *et al*. Enhanced strength and crack resistance in CoCrNi-based medium entropy alloy with nano-precipitates, 9R structures and nanotwins produced by hot isostatic pressing. *Intermetallics* **159**, 107929 (2023).
3. Atkinson, H. V. *et al*. Fundamental aspects of Hot isostatic pressing: an overview. *Metall. Mater. Trans. A* **31**, 2982 (2000).
4. Tran, M.T. *et al*. Effect of hot isostatic pressing on the cryogenic mechanical properties of CrCoNi medium entropy alloy processed by direct energy deposition. *Mater. Sci. Eng* **828**, 142110 (2021).
5. Joseph J. *et al*. Mater Sci Eng 2018;733;59-70). Effect of hot isostatic pressing on the microstructure and mechanical properties of additive manufactured $Al_xCoCrFeNi$ high entropy alloys. *Mater. Sci. Eng.* **733**, 59-70 (2018).
6. Zhang, M. *et al.* On the damage tolerance of 3-D printed Mg-Ti interpenetrating-phase composites with bioinspired architectures. *Nat. Commun.* **13**, 3247 (2022).
7. Wang, H. *et al.* Manufacture-friendly nanostructured metals stabilized by dual-phase honeycomb shell. *Nat. Commun.* **13**, 2034 (2022).
8. Yang, T. *et al.* Ultrahigh-strength and ductile superlattice alloys with nanoscale disordered interfaces. *Science* **369**, 427-432 (2020).
9. Li, X. Y., Jin, Z. H., Zhou, X. & Lu, K. Constrained minimal-interface structures in polycrystalline copper with extremely fine grains. *Science* **370**, 831-836 (2020).
10. Joseph, J. *et al*. Understanding the mechanical behaviour and the large strength/ductility differences between FCC and BCC $Al_xCoCrFeNi$ high entropy alloys. *J. Alloys Compd.* **726**, 885-895 (2017).
11. Zhang, D. D., Zhang, J. Y., Kuang, J., Liu, G. & Sun, J. The B2 phase-driven microstructural heterogeneities and twinning enable ultrahigh cryogenic strength and large ductility in NiCoCr-based medium-entropy alloy. *Acta Mater.* **233**, 117981 (2022).
12. Ateba Betanda, Y. *et al.* Measurement of stored energy in Fe–48%Ni alloys strongly cold-rolled using three approaches: Neutron diffraction, Dillamore and KAM approaches. *Mater. Sci. Eng.* **614**, 193-198 (2014).
13. Zaiser, M. & Aifantis, E. C. Geometrically necessary dislocations and strain gradient plasticity - a dislocation dynamics point of view. *Scr. Mater.* **48**, 133-139 (2003).
14 Gao, H., Huang, Y., Nix, W. D. & Hutchinson, J. W. Mechanism-based strain gradient plasticity - I. Theory. *J. Mech. Phys. Solids* **47**, 1239-1263 (1999).
15. Zhao, Y. L. *et al.* Anomalous precipitate-size-dependent ductility in multicomponent high-entropy alloys with dense nanoscale precipitates. *Acta Mater.* **223**, 117480 (2022).
16. Yang, T. *et al.* Multicomponent intermetallic nanoparticles and superb mechanical behaviors of complex alloys. *Science* **362**, 933-937 (2018).
17. Choudhuri, D., Banerjee, R. & Srinivasan, S. G. Uniaxial deformation of face-centered-cubic(Ni)-ordered B2(NiAl) bicrystals: atomistic mechanisms near a Kurdjumov-Sachs interface. *J. Mater. Sci.* **53**, 5684-5695 (2018).

18. Yang, Y. M., Jeng, S. M., Bain, K. & Amato, R. A. Microstructure and mechanical behavior of in-situ directional solidified NiAl/Cr(Mo) eutectic composite. *Acta Mater.* **45**, 295-308 (1997).

19 Lu, Y. *et al.* Directly cast bulk eutectic and near-eutectic high entropy alloys with balanced strength and ductility in a wide temperature range. *Acta Mater.* **124**, 143-150 (2017).

20 Liu, K. *et al.* Effect of nano-sized precipitates on the fatigue property of a lamellar structured high entropy alloy. *Mater. Sci. Eng.* **760**, 225-230 (2019).

21 Wu, Q. *et al.* Phase-selective recrystallization makes eutectic high-entropy alloys ultra-ductile. *Nat. Commun.* **13**, 4697 (2022).

22 Wei, Q. Q. *et al.* Metal-carbide eutectics with multiprincipal elements make superrefractory alloys. *Sci. Adv.* **8**, eabo2068 (2022).

23 Liu, W. H. *et al.* Effects of Nb additions on the microstructure and mechanical property of CoCrFeNi high-entropy alloys. *Intermetallics* **60**, 1-8 (2015).

24 Williamson, G. K. *et al.* X-ray line broadening from filed aluminium and wolfram. *Acta Metall. Mater.* **1**, 22-31 (1953).

25 Ming, K., Li, L., Li, Z., Bi, X. & Wang, J. Grain boundary decohesion by nanoclustering Ni and Cr separately in CrMnFeCoNi high-entropy alloys. *Sci. Adv.* **5**, eaay0639 (2019).

26 Song, R., Ponge, D., Raabe, D. & Kaspar, R. Microstructure and crystallographic texture of an ultrafine grained C-Mn steel and their evolution during warm deformation and annealing. *Acta Mater.* **53**, 845-858 (2005).

27 Ishida, K. Effect of grain size on grain boundary segregation. *J. Alloys Compd.* **235**, 244-249 (1996).

28 Nyakiti, L. O., Chaudhuri, J. & Jankowski, A. F. High-resolution electron microscopy characterization of nanocrystalline grain boundaries in gold-copper alloys. *Thin Solid Films* **517**, 1182-1185 (2008).

29. Chen, Y. *et al.* Effects of lanthanum addition on microstructure and mechanical properties of as-cast pure copper. *J. Rare Earths* **32**, 1056-1063 (2014).

30 Xiong, T. *et al.* Faceted Kurdjumov-Sachs interface-induced slip continuity in the eutectic high-entropy alloy, $AlCoCrFeNi_{2.1}$. *J. Mater. Sci. Technol.* **65**, 216-227 (2021).

31 Garg, A., et al. Dissociated and faceted large-angle coincident-site-lattice boundaries in silicon. *Philos. Mag. A* **59**, 479-499 (1989).

32 Wang, Y., Chen, M., Zhou, F. & Ma, E. High tensile ductility in a nanostructured metal. *Nature* **419**, 912-915 (2002).

33 Wang, Y. M. *et al.* Additively manufactured hierarchical stainless steels with high strength and ductility. *Nat. Mater.* **17**, 63-71 (2018).

34 Lu, K. Making strong nanomaterials ductile with gradients. *Science* **345**, 1455-1456 (2014).

35 Huang, H. *et al.* Phase-Transformation Ductilization of Brittle High-Entropy Alloys via Metastability Engineering. *Adv. Mater.* **29**, 1701678 (2017).

36 Ma, E. & Zhu, T. Towards strength-ductility synergy through the design of heterogeneous nanostructures in metals. *Mater. Today* **20**, 323-331 (2017).

37 Fang, T. H., Li, W. L., N. R. Tao, Lu, K. Revealing extraordinary intrinsic tensile plasticity in gradient nano-grained copper. *Science* **331**, 1587 (2011).
